# Supplementary figures and images for: Community- and trophic-level responses of soil nematodes to removal of a non-native tree at different stages of invasion
Source: PLoS One. 2020 Jan 10;15(1):e0227130. doi: 10.1371/journal.pone.0227130 (PMC6953854; doi:10.1371/journal.pone.0227130)

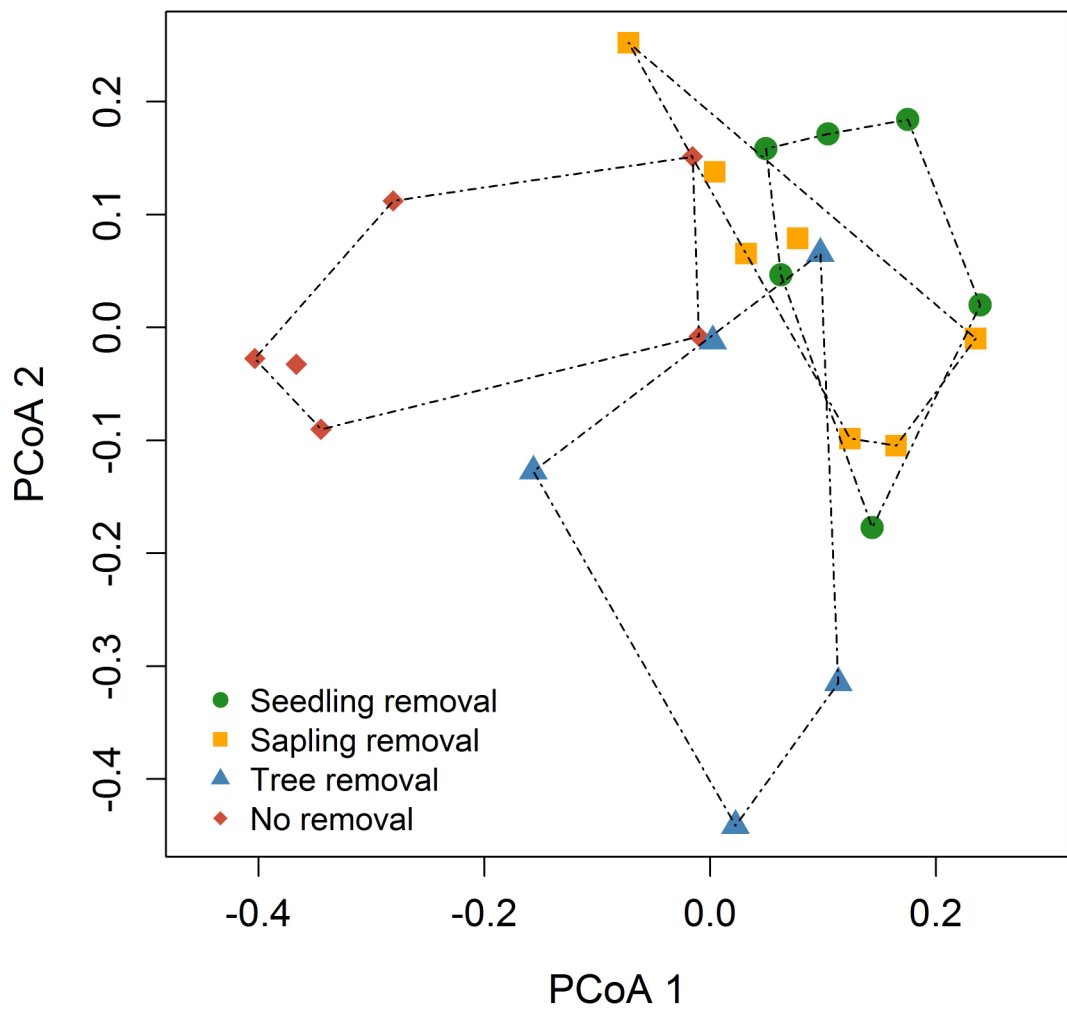

Supplement: S1 Fig — Management strategies representing different stages of invasion process: seedling removal, sapling removal, tree removal, no removal. Principal Coordinate analyses were based on the Jaccard dissimilarity metric. Sites closer together in multivariate space have similar compositions. Dashed lines represent convex hulls in ordination space. (PDF) [file pone.0227130.s001.pdf]

A

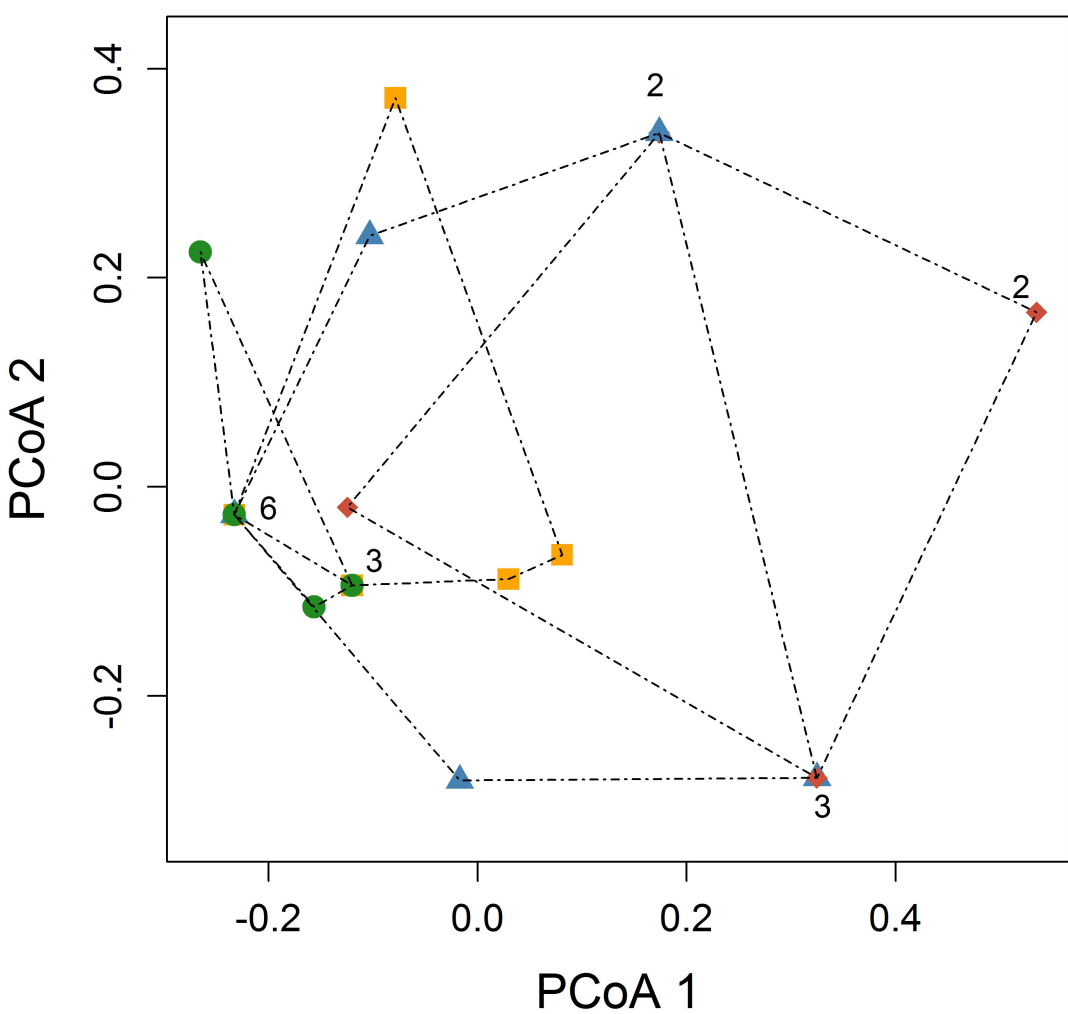

B

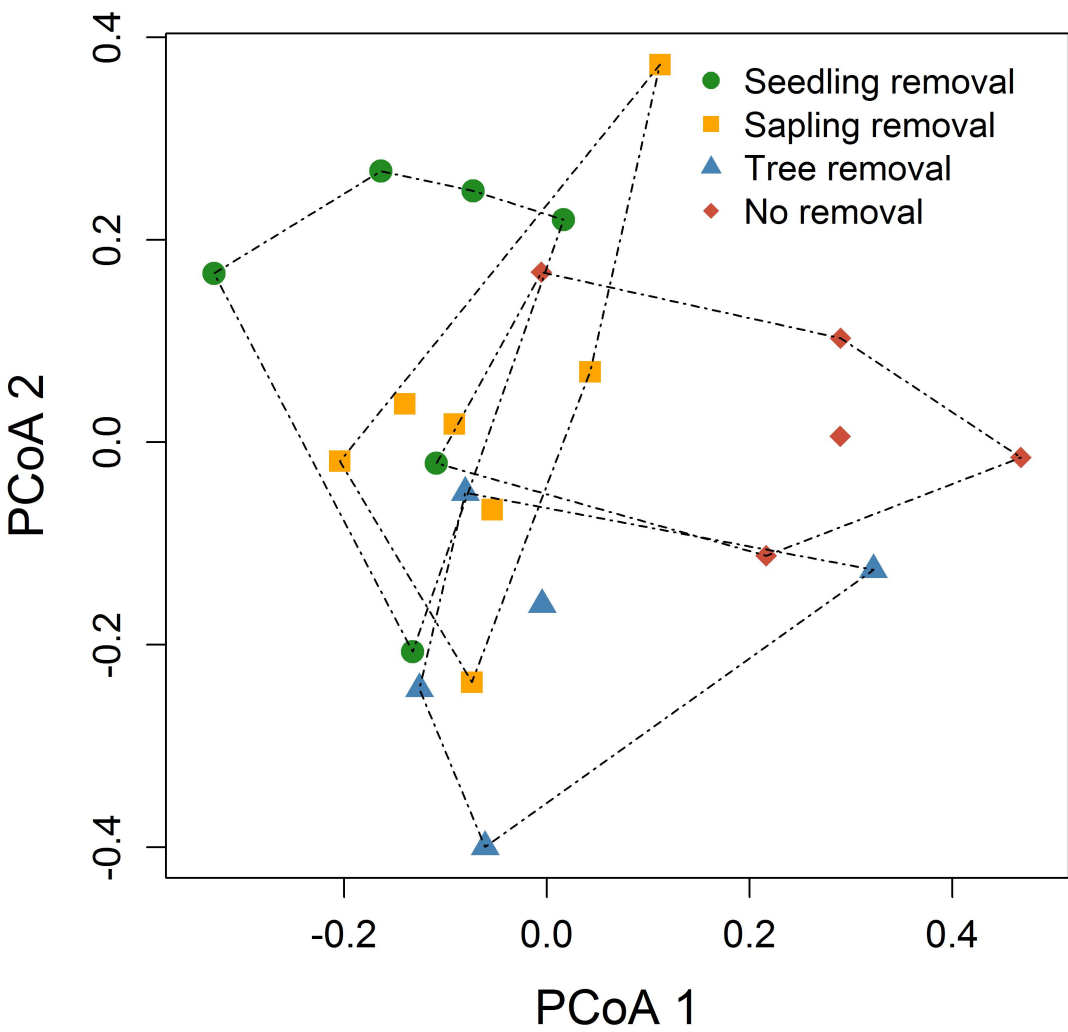

C

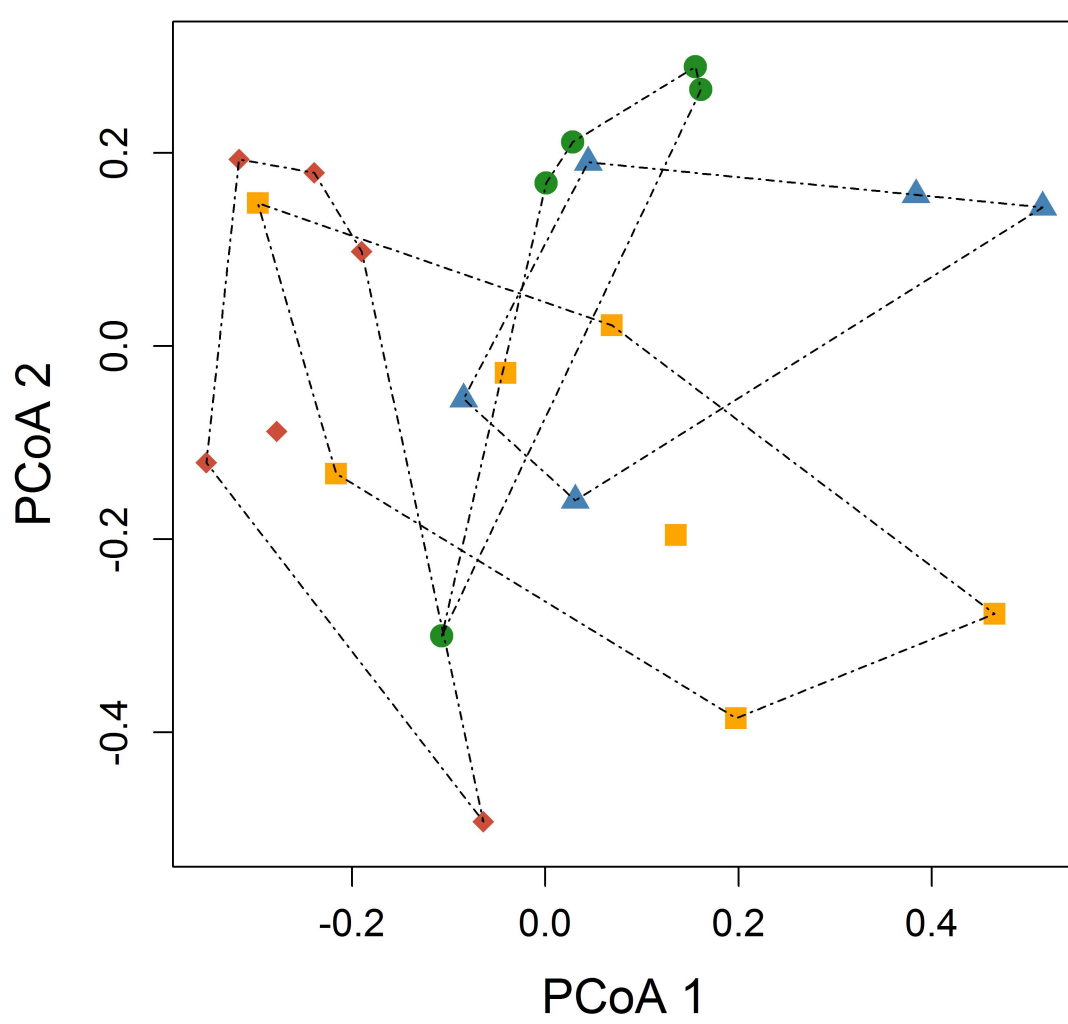

Supplement: S2 Fig — Trophic levels: (A) TL1, (B) TL2, (B) TL3. Management strategies: seedling removal, sapling removal, tree removal, no removal. Principal Coordinate analyses were based on the Jaccard dissimilarity metric. Sites closer together in multivariate space have more similar compositions. (PDF) [file pone.0227130.s002.pdf]
