# Supplementary material for: Community- and trophic-level responses of soil nematodes to removal of a non-native tree at different stages of invasion
Source: PLoS One. 2020 Jan 10;15(1):e0227130. doi: 10.1371/journal.pone.0227130 (PMC6953854; doi:10.1371/journal.pone.0227130)
Supplement: S1 Table — Nematode taxon, feeding group (plant feeder, plant associated, bacterial feeder, fungal feeder, predator, omnivore), trophic level (1–3) to which each taxa was assigned and management strategy where each taxa was present (U = seedling removal, SR = sapling removal, TR = tree removal, NR = No removal). (DOCX) [file pone.0227130.s003.docx]

**S1 Table**

|  | **Family** | **Taxa** | **Feeding group** | **Trophic level** | **Presence** |
| --- | --- | --- | --- | --- | --- |
| 1 | Cephalobidae | Acrobeloides | Bacterial feeder | 2 | U |
| 2 | Alaimidae | Alaimus sp 1 | Bacterial feeder | 2 | U, SR, TR |
| 3 | Alaimidae | Alaimus sp 2 | Bacterial feeder | 2 | U, SR, NR |
| 4 | Plectidae | Anaplectus | Bacterial feeder | 2 | U |
| 5 | Aphelenchoididae | Aphelenchoididae | Fungal feeder | 2 | U, SR, NR |
| 6 | Aporcelaimidae | Aporcelaimidae | Omnivore | 3 | U, SR, NR |
| 7 | Belondiridae | Belondiridae | Plant associated | 1 | U, SR, NR |
| 8 | Tylenchidae | Cephalenchus | Plant associated | 1 | U, SR, TR, NR |
| 9 | Cephalobidae | Cephalobus | Bacterial feeder | 2 | U, SR, TR, NR |
| 10 | Cephalobidae | Cervidellus | Bacterial feeder | 2 | U, SR, TR, NR |
| 11 | Chromadoridae | Chromadoridae sp M | Bacterial feeder | 2 | U, SR, TR, NR |
| 12 | Chromadoridae | Chromadoridae sp P | Predator | 3 | SR, TR, NR |
| 13 | Mononchidae | Clarkus | Predator | 3 | U, SR, TR, NR |
| 14 | Mononchidae | Cobbonchus | Predator | 3 | TR |
| 15 | Criconematidae | Criconematid | Plant feeder | 1 | U, SR, TR, NR |
| 16 | Diphtherophoridae | Diphtherophora | Fungal feeder | 2 | SR, NR |
| 17 | Anguinidae | Ditylenchus | Fungal feeder | 2 | U, SR, TR, NR |
| 18 | Dorylaimidae | Dorylaimus sp 1 | Omnivore | 3 | SR |
| 19 | Dorylaimidae | Dorylaimus sp 2 | Omnivore | 3 | SR |
| 20 | Belondiridae | Dorylaimellus | Plant associated | 1 | U, SR, TR |
| 21 | Leptonchidae | Doryllium | Fungal feeder | 2 | U, SR |
| 22 | Dorylaimidae | Eudorylaimus sp 1 | Omnivore | 3 | U, SR, TR, NR |
| 23 | Dorylaimidae | Eudorylaimus sp 2 | Omnivore | 3 | U, SR, TR |
| 24 | Dorylaimidae | Eudorylaimus sp 3 | Omnivore | 3 | SR |
| 25 | Dorylaimidae | Eudorylaimus sp 4 | Omnivore | 3 | U |
| 26 | Monhysteridae | Geomonhystera | Bacterial feeder | 2 | U, TR |
| 27 | Hoplolaimidae | Hoplolaimidae | Plant feeder | 1 | SR |
| 28 | Mononchidae | Iotonchus | Predator | 3 | U, TR |
| 29 | Qudsianematidae | Labronema | Omnivore | 3 | U, SR, TR, NR |
| 30 | Monhysteridae | Monhystera | Bacterial feeder | 2 | U, SR, TR, NR |
| 31 | Mononchidae | Mylonchulus | Predator | 3 | SR, NR |
| 32 | Nygolaimidae | Nygolaimus sp 1 | Predator | 3 | U, SR, TR, NR |
| 33 | Panagrolaimidae | Panagrolaimus | Bacterial feeder | 2 | TR, NR |
| 34 | Tylenchulidae | Paratylenchus | Plant feeder | 1 | U, SR, TR, NR |
| 35 | Plectidae | Plectus_delic | Bacterial feeder | 2 | U, SR, TR |
| 36 | Plectidae | Plectus_robus | Bacterial feeder | 2 | U, SR, TR, NR |
| 37 | Mononchidae | Prionchulus | Predator | 3 | TR |
| 38 | Prismatolaimidae | Prismatolaimus | Bacterial feeder | 2 | U, SR, TR, NR |
| 39 | Rhabditidae | Rhabditidae | Bacterial feeder | 2 | U, SR, TR, NR |
| 40 | Rhabdolaimidae | Rhabdolaimus | Bacterial feeder | 2 | U, SR, TR, NR |
| 41 | Aporcelaimidae | Sectonema | Predator | 3 | SR, NR |
| 42 | Teratocephalidae | Teratocephalus | Bacterial feeder | 2 | U, SR, TR, NR |
| 43 | Tylencholaimidae | Tylencholaimus sp 2 | Fungal feeder | 2 | U, TR |
| 44 | Tylenchidae | Tylenchus sp 1 | Plant associated | 1 | U, SR, TR, NR |
| 45 | Tylenchidae | Tylenchus sp 2 | Plant associated | 1 | U, SR, TR, NR |
| 46 | Plectidae | Wilsonema | Bacterial feeder | 2 | U, SR, TR, NR |
