# Supplementary material for: Community- and trophic-level responses of soil nematodes to removal of a non-native tree at different stages of invasion
Source: PLoS One. 2020 Jan 10;15(1):e0227130. doi: 10.1371/journal.pone.0227130 (PMC6953854; doi:10.1371/journal.pone.0227130)
Supplement: S2 Table — TL = trophic level. Bold values indicate significant results (α = 0.05). (DOCX) [file pone.0227130.s004.docx]

**S2 Table**

| **Response variable** | **Fixed effects** | **Estimate ± SE** | **Z-value** | **P** |
| --- | --- | --- | --- | --- |
| Total taxa richness | Intercept | 2.936 ± 0.094 | 31.206 | **<0.001** |
|  | Sapling removal | -0.061 ± 0.130 | -0.471 | 0.638 |
|  | No removal | -0.358 ± 0.147 | -2.441 | **0.015** |
|  | Tree removal | -0.163 ± 0.146 | -1.116 | 0.264 |
| TL 1 taxa richness | Intercept | 1.575 ± 0.186 | 8.485 | **<0.001** |
|  | Sapling removal | -0.154 ± 0.263 | -0.587 | 0.557 |
|  | No removal | -0.802 ± 0.334 | -2.404 | **0.016** |
|  | Tree removal | -0.412 ± 0.311 | -1.324 | 0.185 |
| TL 2 taxa richness | Intercept | 2.251 ± 0.132 | 16.997 | **<0.001** |
|  | Sapling removal | -0.103 ± 0.185 | -0.556 | 0.578 |
|  | No removal | -0.193 ± 0.197 | -0.979 | 0.328 |
|  | Tree removal | -0.123 ± 0.203 | -0.605 | 0.545 |
| TL 3 taxa richness | Intercept | 1.504 ± 0.192 | 7.815 | **<0.001** |
|  | Sapling removal | 0.105 ± 0.256 | 0.411 | 0.681 |
|  | No removal | -0.351 ± 0.299 | -1.173 | 0.241 |
|  | Tree removal | -0.022 ± 0.287 | -0.078 | 0.938 |
