# Supplementary material for: Community- and trophic-level responses of soil nematodes to removal of a non-native tree at different stages of invasion
Source: PLoS One. 2020 Jan 10;15(1):e0227130. doi: 10.1371/journal.pone.0227130 (PMC6953854; doi:10.1371/journal.pone.0227130)
Supplement: S3 Table — Management strategies: seedling removal, sapling removal, no removal, tree removal. Bold values indicate significant results (α = 0.05). (DOCX) [file pone.0227130.s005.docx]

**S3 Table**

| **Dissimilarity metric** | **Community composition** | **Source** | **df** | **SS** | **MS** | **Pseudo-F** | **P-value** |
| --- | --- | --- | --- | --- | --- | --- | --- |
| Jaccard | All nematodes | Management strategies | 3 | 0.935 | 0.312 | 2.039 | **<0.001** |
|  |  | Residuals | 20 | 3.058 | 0.153 |  |  |
|  |  | Total | 23 | 3.993 |  |  |  |
| Jaccard | TL 1 | Management strategies | 3 | 0.933 | 0.311 | 2.761 | **0.008** |
|  |  | Residuals | 20 | 2.251 | 0.112 |  |  |
|  |  | Total | 23 | 3.183 |  |  |  |
| Jaccard | TL 2 | Management strategies | 3 | 0.835 | 0.278 | 1.729 | **0.012** |
|  |  | Residuals | 20 | 3.221 | 0.161 |  |  |
|  |  | Total | 23 | 4.057 |  |  |  |
| Jaccard | TL 3 | Management strategies | 3 | 1.165 | 0.388 | 2.010 | **0.006** |
|  |  | Residuals | 20 | 3.863 | 0.193 |  |  |
|  |  | Total | 3 | 5.028 |  |  |  |
| Bray-Curtis | All nematodes | Management strategies | 3 | 1.385 | 0.461 | 3.334 | **<0.001** |
|  |  | Residuals | 20 | 2.769 | 0.138 |  |  |
|  |  | Total | 23 | 4.153 |  |  |  |
| Bray-Curtis | TL 1 | Management strategies | 3 | 1.255 | 0.418 | 3.464 | **<0.001** |
|  |  | Residuals | 20 | 2.416 | 0.121 |  |  |
|  |  | Total | 23 | 3.671 |  |  |  |
| Bray-Curtis | TL 2 | Management strategies | 3 | 1.542 | 0.514 | 3.714 | **<0.001** |
|  |  | Residuals | 20 | 2.769 | 0.138 |  |  |
|  |  | Total | 23 | 4.311 |  |  |  |
| Bray-Curtis | TL 3 | Management strategies | 3 | 0.964 | 0.321 | 1.639 | **0.050** |
|  |  | Residuals | 20 | 3.922 | 0.196 |  |  |
|  |  | Total | 23 | 4.887 |  |  |  |
