# Supplementary material for: Community- and trophic-level responses of soil nematodes to removal of a non-native tree at different stages of invasion
Source: PLoS One. 2020 Jan 10;15(1):e0227130. doi: 10.1371/journal.pone.0227130 (PMC6953854; doi:10.1371/journal.pone.0227130)
Supplement: S4 Table — Management strategies: seedling removal, sapling removal, no removal, tree removal. Jaccard and Bray-Curtis dissimilarity metrics were used to assess differences in community composition. Bold values indicate significant differences in community composition (α = 0.05). (DOCX) [file pone.0227130.s006.docx]

**S4 Table**

| **Dissimilarity metric** | **Community composition** | **Management strategies pairs** | **Pseudo-F** | **P-value** |
| --- | --- | --- | --- | --- |
| Jaccard | All nematodes | No removal vs. Sapling removal | 2.397 | **0.013** |
|  |  | No removal vs. Tree removal | 2.064 | **0.017** |
|  |  | No removal vs. Seedling removal | 4.147 | **0.002** |
|  |  | Sapling removal vs. Tree removal | 1.194 | 0.269 |
|  |  | Sapling removal vs. Seedling removal | 1.126 | 0.332 |
|  |  | Tree removal vs. Seedling removal | 1.653 | 0.085 |
| Jaccard | TL 1 | No removal vs. Sapling removal | 4.303 | **0.010** |
|  |  | No removal vs. Tree removal | 1.489 | 0.247 |
|  |  | No removal vs. Seedling removal | 7.487 | **0.006** |
|  |  | Sapling removal vs. Tree removal | 0.707 | 0.633 |
|  |  | Sapling removal vs. Seedling removal | 1.241 | 0.334 |
|  |  | Tree removal vs. Seedling removal | 1.404 | 0.261 |
| Jaccard | TL 2 | No removal vs. Sapling removal | 1.580 | 0.098 |
|  |  | No removal vs. Tree removal | 1.548 | 0.175 |
|  |  | No removal vs. Seedling removal | 3.006 | **0.007** |
|  |  | Sapling removal vs. Tree removal | 1.365 | 0.199 |
|  |  | Sapling removal vs. Seedling removal | 0.980 | 0.466 |
|  |  | Tree removal vs. Seedling removal | 2.110 | **0.048** |
| Jaccard | TL 3 | No removal vs. Sapling removal | 2.525 | **0.026** |
|  |  | No removal vs. Tree removal | 3.290 | **0.002** |
|  |  | No removal vs. Seedling removal | 3.519 | **0.013** |
|  |  | Sapling removal vs. Tree removal | 1.061 | 0.396 |
|  |  | Sapling removal vs. Seedling removal | 1.512 | 0.187 |
|  |  | Tree removal vs. Seedling removal | 0.633 | 0.744 |
| Bray-Curtis | All nematodes | No removal vs. Sapling removal | 4.622 | **0.002** |
|  |  | No removal vs. Tree removal | 3.641 | **0.005** |
|  |  | No removal vs. Seedling removal | 9.098 | **0.003** |
|  |  | Sapling removal vs. Tree removal | 2.037 | **0.020** |
|  |  | Sapling removal vs. Seedling removal | 0.843 | 0.688 |
|  |  | Tree removal vs. Seedling removal | 2.234 | **0.009** |
| Bray-Curtis | TL 1 | No removal vs. Sapling removal | 4.031 | **0.002** |
|  |  | No removal vs. Tree removal | 2.151 | 0.090 |
|  |  | No removal vs. Seedling removal | 10.123 | **0.002** |
|  |  | Sapling removal vs. Tree removal | 1.858 | 0.080 |
|  |  | Sapling removal vs. Seedling removal | 1.310 | 0.209 |
|  |  | Tree removal vs. Seedling removal | 3.330 | **0.006** |
| Bray-Curtis | TL 2 | No removal vs. Sapling removal | 5.657 | **0.005** |
|  |  | No removal vs. Tree removal | 4.807 | **0.004** |
|  |  | No removal vs. Seedling removal | 9.512 | **0.007** |
|  |  | Sapling removal vs. Tree removal | 2.248 | **0.030** |
|  |  | Sapling removal vs. Seedling removal | 0.730 | 0.726 |
|  |  | Tree removal vs. Seedling removal | 2.058 | 0.065 |
| Bray-Curtis | TL 3 | No removal vs. Sapling removal | 2.841 | **0.011** |
|  |  | No removal vs. Tree removal | 2.238 | **0.048** |
|  |  | No removal vs. Seedling removal | 3.866 | **0.009** |
|  |  | Sapling removal vs. Tree removal | 0.848 | 0.592 |
|  |  | Sapling removal vs. Seedling removal | 0.565 | 0.831 |
|  |  | Tree removal vs. Seedling removal | 0.625 | 0.709 |
