# Supplementary material for: Community- and trophic-level responses of soil nematodes to removal of a non-native tree at different stages of invasion
Source: PLoS One. 2020 Jan 10;15(1):e0227130. doi: 10.1371/journal.pone.0227130 (PMC6953854; doi:10.1371/journal.pone.0227130)
Supplement: S5 Table — Significant indicator taxa of individual or group of management strategies as identified by indicator species analyses for each trophic level. Analyses were performed including taxa abundance and using only presence-absence data. P values were obtained by permuting the data 9999 times. Bold values indicate significant results (α = 0.05). (DOCX) [file pone.0227130.s007.docx]

**S5 Table**

| **Data** | **Trophic level** | **Management strategy or groups of management strategies** | **Taxa** | **stat** | **P** |
| --- | --- | --- | --- | --- | --- |
| Nematode abundance | TL 1 | Seedling removal + sapling removal | Criconematid | 0.951 | 0.002 |
|  |  | Seedling removal + sapling removal +  tree removal | Dorylaimellus | 0.85 | 0.020 |
|  | TL 2 | Seedling removal | Doryllium | 0.665 | 0.026 |
|  |  | Seedling removal + sampling removal +  tree removal | Tylencholaimus sp 2 | 0.901 | 0.016 |
|  |  | Sapling removal +  tree removal +  no removal | Plectus robus | 0.910 | 0.018 |
|  | TL 3 | Seedling removal + sapling removal +  tree removal | Aporcelaimidae | 0.913 | 0.003 |
| Nematode presence-abscence | TL 1 | Seedling removal + sapling removal +  tree removal | Criconematid | 0.888 | 0.042 |
|  |  |  | Dorylaimellus | 0.850 | 0.009 |
|  | TL 2 | Sapling removal +  tree removal +  no removal | Plectus robus | 0.890 | 0.015 |
|  | TL 3 | Seedling removal + sapling removal +  tree removal | Aporcelaimidae | 0.913 | 0.002 |
