# Supplementary material for: Community- and trophic-level responses of soil nematodes to removal of a non-native tree at different stages of invasion
Source: PLoS One. 2020 Jan 10;15(1):e0227130. doi: 10.1371/journal.pone.0227130 (PMC6953854; doi:10.1371/journal.pone.0227130)
Supplement: Attachment — Submitted filename: Response to Reviewers.pdf [file pone.0227130.s009.pdf]

Response to reviewers

Additional Editor Comments (if provided):

The manuscript is much improved. There are only few minor comments. However, I think some changes to the new Figure 4 are warranted prior to publication. In its current form, it is not immediately intuitive how the data are depicted.
>> We appreciate the Editor and reviewers' positive feedback and comments. We have incorporated all the comments made by the reviewers (including a modified version of Figure 4) and responded specifically to each comment below, marking our responses with '>>'.

Reviewers' comments:
Reviewer's Responses to Questions

Comments to the Author

1. If the authors have adequately addressed your comments raised in a previous round of review and you feel that this manuscript is now acceptable for publication, you may indicate that here to bypass the "Comments to the Author" section, enter your conflict of interest statement in the "Confidential to Editor" section, and submit your "Accept" recommendation.
Reviewer #1: (No Response)
Reviewer #2: All comments have been addressed
>> We have modified Figure 4 and incorporated all the minor comments suggested by Reviewer #1.

2. Is the manuscript technically sound, and do the data support the conclusions?
The manuscript must describe a technically sound piece of scientific research with data that supports the conclusions. Experiments must have been conducted rigorously, with appropriate controls, replication, and sample sizes. The conclusions must be drawn appropriately based on the data presented.
Reviewer #1: Yes
Reviewer #2: Yes

3. Has the statistical analysis been performed appropriately and rigorously?
Reviewer #1: Yes
Reviewer #2: Yes

4. Have the authors made all data underlying the findings in their manuscript fully available?
The PLOS Data policy requires authors to make all data underlying the findings described in their manuscript fully available without restriction, with rare exception (please refer to the Data Availability Statement in the manuscript PDF file). The data should be provided as part of the manuscript or its supporting information, or deposited to a public repository. For example, in addition to summary statistics, the data points behind means, medians and variance measures should be available. If there are restrictions on publicly sharing data—e.g. participant privacy or use of data from a third party—those must be specified.

Reviewer #1: Yes
Reviewer #2: Yes

5. Is the manuscript presented in an intelligible fashion and written in standard English?
PLOS ONE does not copyedit accepted manuscripts, so the language in submitted articles must be clear, correct, and unambiguous. Any typographical or grammatical errors should be corrected at revision, so please note any specific errors here.
Reviewer #1: Yes
Reviewer #2: Yes

6. Review Comments to the Author
Please use the space provided to explain your answers to the questions above. You may also include additional comments for the author, including concerns about dual publication, research ethics, or publication ethics. (Please upload your review as an attachment if it exceeds 20,000 characters)

Reviewer #1: I appreciate the efforts of the authors to revise this manuscript. The manuscript is much improved, particularly regarding the new analyses and restructuring of the Introduction and Discussion. The authors have highlighted the original Dickie et al. 2014 paper in AoB Plants, and how this manuscript builds upon that published work. Additionally, the authors provide greater methodological details and justification for the tree, sapling, and seedling removal management treatments. Specific plans for making the data publicly available have now been stated, complying with the PLOS data policy. I have only a few concerns to be addressed, outlined below.

Results:

S2 Table: Table caption isn't overly informative. This table appears to summarize results comparing the seedling removal treatment to the other treatments, so should be stated in the table caption.
>> We have changed the S2 Table caption as suggested by the reviewer. It now reads: "S2 Table. Results of generalised linear models with Poisson error distribution comparing taxa richness of the seedling removal management strategy with taxa richness of the sapling removal, no removal and tree removal management strategies. TL = trophic level. Bold values indicate significant results ($\alpha = 0.05$)."

Figure 4: I'm a bit confused by this figure. For relative abundance data, figures are normally shown in percentages. Since an unequal number of nematodes were identified per sample ('approximately 100'), standardizing those to values to percentages would be most appropriate. A quick glance at Figure 4 reveals that adding up the bars of relative abundance for T1, T2, and T3 do not appear to sum to 100. That makes it harder to attribute differences to treatments versus sampling effort. It may be worthwhile to offer a more thorough explanation in the figure legend as well.
>> We appreciate the comment and apologise for the imprecision. We have now modified Figure 4 following the reviewer suggestion. Specifically, we replaced total abundances by percentages as suggested by the reviewer. We have also expanded the figure legend to clarify that the figure shows the percentage composition of each nematode taxon within a sampling site across management strategies and trophic levels.

Minor comments:
Line 78: change ‘removal or…’ to ‘removal of…’
>> We have changed this as suggested.

Line 329: I think you mean ‘were’ not ‘where’
>> We have changed this as suggested.

Line 350: change ‘completed’ to ‘completely’
>> We have changed this as suggested.

Reviewer #2: The authors have considered my and other reviewer comments and made satisfactory and considerable changes in the new version of the manuscript "Community- and trophic-level responses of soil nematodes to removal of a non-native tree at different stages of invasion". The text has been edited significantly and the readability and flow of the manuscript has greatly improved. The change of an informative title and the reanalysis of the data, the addition of associated visual elements have resulted in a more interesting manuscript. Overall, this manuscript has been improved significantly.
>> We appreciate the positive appraisal of our manuscript.
